# Markov Models of Use-Dependence and Reverse Use-Dependence during the Mouse Cardiac Action Potential

**DOI:** 10.1371/journal.pone.0042295

**Published:** 2012-08-06

**Authors:** Qinlian Zhou, Glenna C. L. Bett, Randall L. Rasmusson

**Affiliations:** 1 Center for Cellular and Systems Electrophysiology, State University of New York, University at Buffalo, Buffalo, New York, United States of America; 2 Physiology and Biophysics, State University of New York, University at Buffalo, Buffalo, New York, United States of America; 3 Biomedical Engineering, State University of New York, University at Buffalo, Buffalo, New York, United States of America; 4 Gynecology-Obstetrics, State University of New York, University at Buffalo, Buffalo, New York, United States of America; Baylor College of Medicine, United States of America

## Abstract

The fast component of the cardiac transient outward current, I_Ktof_, is blocked by a number of drugs. The major molecular bases of I_Ktof_ are Kv4.2/Kv4.3 voltage-gated potassium channels. Drugs with similar potencies but different blocking mechanisms have differing effects on action potential duration (APD). We used *in silico* analysis to determine the effect of I_Ktof_-blocking drugs with different blocking mechanisms on mouse ventricular myocytes. We used our existing mouse model of the action potential, and developed 4 new Markov formulations for I_Ktof_, I_Ktos_, I_Kur_, I_Ks_. We compared effects of theoretical I_Ktof_-specific channel blockers: (1) a closed state, and (2) an open channel blocker. At concentrations lower or close to IC_50_, the drug which bound to the open state always had a much greater effect on APD than the drug which bound to the closed state. At concentrations much higher than IC_50_, both mechanisms had similar effects at very low pacing rates. However, an open state binding drug had a greater effect on APD at faster pacing rates, particularly around 10 Hz. In summary, our data indicate that drug effects on APD are strongly dependent not only on IC_50_, but also on the drug binding state.

## Introduction

Delayed cardiac repolarization and the associated prolongation of the QT interval on the EKG is an undesired side effect of many drugs [1]–[3]. This includes drugs whose intended therapeutic effect is on an organ other than the heart. Following the withdrawal of a number of non-cardiovascular drugs that had their label revised or were completely withdrawn from the market because of cardiac safety issues [4], the FDA now requires all novel therapeutic drugs to pass QT safety screening [5]. Consequently, it is of great importance to develop new tools and methods that can identify, as early as possible, the risk of novel agents in arrhythmogenesis. This requires in depth understanding of the mechanisms by which drug-induced modifications of normal ion channel behavior lead to cardiac arrhythmias.

One of the critical factors in drug binding is the consequences of conformation-dependent binding of the blocker to the channel, i.e., the kinetics of drug-channel interaction. This is difficult to analyze. Furthermore, because multiple overlapping channels interact with each other during the action potential (AP), it is difficult to interpret effects of drugs on AP morphology and restitution (i.e., action potential excitability and duration change in response to increasing prematurity of stimulation). Changes in restitution are an important index of the likelihood of arrhythmic behavior [6]. Molecularly based mathematical models offer a unique insight into the putative consequences of state-dependent drug binding on repolarization and restitution. Markov models are particularly useful when simulating drug-channel interactions, as alteration of the kinetic properties of a single ion channel caused by drug binding can be related to changes in specific rate constants in the Markov model.

We have developed a mathematical model of the mouse ventricular myocyte consisting of solely Markov type models, and based on voltage clamp electrophysiological data. We developed models of the mouse endocardial and epicardial ventricular myocyte, and used them to predict the kinetic consequences of pharmacological modifications on channel properties on the AP. Starting from the endocardial and epicardial models we developed previously [7], we changed four voltage-gated potassium currents from Hodgkin-Huxley (HH) type to Markov models. The four channels modified were: the rapidly inactivating transient outward current I_Ktof_; the slow-inactivating transient outward current I_Ktos_; the ultra-rapidly activating delayed-rectifier current I_Kur_; and the slow delayed-rectifier current I_Ks_. Our new model has Markov representations for all major ion currents and therefore has the potential to be readily used to study the molecular mechanisms of arrhythmogenesis due to pharmacological interventions or genetic mutations.

Most voltage-gated K^+^ channel blockers exhibit reverse use dependence, i.e., the drug is less effective in prolonging AP duration (APD) as heart rate increases. When the heart rate decreases, for example during bradycardia, K^+^ channel blockers commonly show proarrhythmic behavior. Therefore, the effect of K^+^ channel blockers on APD relies heavily on the heart rate. To understand the rate dependent effects of channel blockers, the following three factors have to be considered:

1. The specificity of the drug for a channel;
2. K^+^ channels change their roles in AP repolarization at different heart rates and the interactions between the channels are complicated and rate dependent as well;
3. There are several mechanisms of drug-channel interactions and channel blockers can exhibit use or reverse use dependence at the single channel level, i.e., they reduce the current more (use dependence) or less (reverse use dependence) at higher rates of stimulation. The rate dependence of a drug under voltage clamp is not necessarily the same as its rate dependence on the action potential at the whole cell level, and it is difficult to draw the connection from one to the other. Our newly-developed AP models predict the effects of a drug on APD at different heart rates, based on experimental drug-channel binding kinetics.

The rapidly inactivating transient outward current, I_Ktof_, is blocked by a number of drugs. Campbell et al [8] studied the effect of 4-AP on I_Ktof_ and demonstrated reverse use dependence. 4-AP is thought to bind to the closed state of I_Ktof_. Conversely, quinidine, another I_Ktof_ blocker, binds to the open state and shows conventional use dependence [9]. We simulated results from two hypothetical I_Ktof_ specific drugs, based on the properties of 4-AP and quinidine. We modeled one drug that bound only to the open state of the I_Ktof_ channel, and one that bound only to the closed states of I_Ktof_. We determined the effect of these two drugs on APD at various concentration and pacing frequencies, as well as on AP restitution.

## Results

We developed distinct epicardial and endocardial models of the mouse cardiac ventricular myocyte, based on our existing model of the mouse heart [7]. We modified the representation of four potassium currents from Hodgkin-Huxley type to Markov type models. Modifications were made to: 1) the rapidly inactivating transient outward K^+^ current I_Ktof_; 2) the slow-inactivating transient outward K^+^ current I_Ktos_; 3) the slow delayed-rectifier K^+^ current I_Ks_; and 4) the ultrarapidly activating delayed rectifier K^+^ current I_Kur_.

1. **The Rapidly Inactivating Transient Outward K^+^ Current I_Ktof_;**

I_Ktof_ is a rapidly activating current which plays a role in early repolarization of the action potential. The molecular basis of this channel is the Kv4.x family [10], [11] The model structure for I_Ktof_ is based on our previous kinetic analysis and experimental data [12] as shown in Scheme 1. The rate constants were adjusted to match experimental mouse data [11], [13].
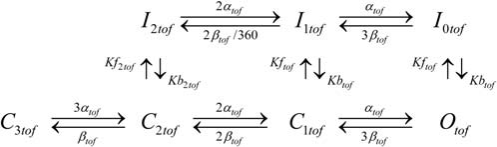
Scheme 1


Current flow is determined according to the following equation: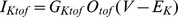.

Where 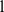 is whole cell conductance, 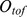 is the probability of the channel being in the open state, 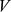
is the membrane potential and 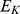 the K^+^ reversal potential. Detailed equations and rate constants are given in Text S1.

The response of the Markov I_Ktof_ model to a standard double-pulse protocol, the peak current-voltage relationship from simulated and experimental data [11], and the comparison of the simulated and experimental steady-state inactivation relationships for I_Ktof_ is shown in **Figure S1**.

In order to compare the results of the Markov model to experimental data and the previous Hodgkin-Huxley type model [7], we used the conductance of 0.4067 ms/uF in model apical cell in this simulation. Similar results were obtained for the epicardial and endocardial cell models, which differ only in the magnitude.

1. **The Slow-inactivating Transient Outward K^+^ Current I_Ktos_;**

The slow inactivating component of the transient outward current is largely encoded by Kv1.4. We have previously derived a model structure for I_Ktos_ [14], as shown in Scheme 3. It has four closed states 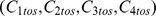, one open state 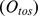, two C-type inactivated states 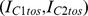, one N-type inactivated state 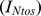, and another inactivated state 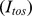 coupling between the C- and N-type inactivation.
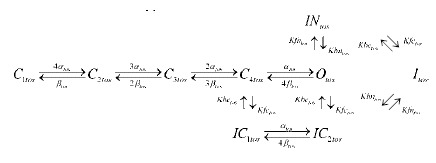
Scheme 2


I_Ktos_ is described by the following equation: 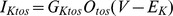, Where 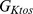 is the whole cell conductance, 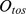
is the probability of the channel being in the open state. *V* is the membrane potential and *E_K_* the K^+^ reversal potential. Equations and rate constants for the model are given in Text S1.

**Figure S2** shows simulated I_Ktos_ current traces in response to step depolarizations, peak I-V relationship, and steady state inactivation relationship compared to experimental data [11]. In order to compare the results of the Markov model to experimental data and our previous Hodgkin-Huxley type model, we used the conductance of 0.0629 ms/uF in model septum cell in this simulation. In the endocardial and epicardial cell models the conductance of I_Ktos_ is set to zero.

### The Slow Delayed-Rectifier K^+^ Current I_Ks_

The molecular basis of I_Ks_ is KCNQ1 and KCNE1 [15]. The model structure for I_Ks_ is from Silva and Rudy [16] as shown in Scheme 3.
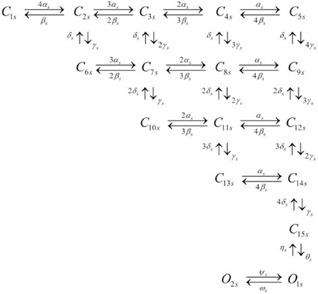
Scheme 3


I_Ks_ is described by the following equation: 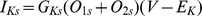 Where *G_Ks_* is the whole cell conductance, 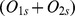
is the probability of the channel being in one of the open states, *V* is the membrane potential, and *E_K_* is the K^+^ reversal potential. Equations and rate constants for the model are given in the Text S1.

### The Ultrarapidly Activating Delayed Rectifier K^+^ Current I_Kur_

The molecular basis of the ultra-rapidly activating delayed rectifier current I_Kur_ is Kv1.5 [17]. Scheme 4 shows the model we used, with four closed states 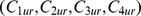, one open state (*O_ur_*), and one inactivated state (*I_ur_*).
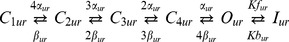
Scheme 4


I_Kur_ is described by the following equation: 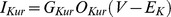 Where *G_Kur_* is the whole cell conductance, *O_ur_* is the probability of the channel being in the open state, *V* is the membrane potential, and *E_K_* the K^+^ reversal potential. Equations and rate constants for the model are given in the Supplementary Information. **Figure S3** shows simulated I_Kur_ current traces in response to step depolarizations, as well as Peak I-V and steady state inactivation relationships.

### Mouse Action Potential

We developed models for epicardial and endocardial mouse ventricular myocytes with all currents represented by Markov models. The differences between epicardial and endocardial cells are different conductances for I_Ktof_, I_Kur_, I_Kss_ and changes in other currents or exchangers as documented in Bondarenko *et al* [7], [18]. The simulated epicardial cell has shorter AP duration (APD) than the endocardial cell, in accordance with our previous modeling [7] and experimental results [19].

### Drug Binding to I_Ktof_

We developed two idealized drugs to investigate the consequences of drugs which bind to only specific ion channel conformations. These hypothetical drugs are idealized versions of two categories of commonly used experimental drugs, closed state blockers (e.g. 4-AP), and open state blockers (e.g., quinidine, clofilium, and long-chain quaternary ammonium compounds) [9], [20], [21]. Previously, we analyzed the block of the calcium-independent transient outward K^+^ current by 4-aminopyridine (4-AP) in ferret ventricular myocytes [8]. 4-AP reduces I_Ktof_ in a reverse use-dependent manner through a closed state binding mechanism [8]. We developed a hypothetical drug (drug C) based on the closed state binding characteristics of 4-AP. The idealized drug competitively binds to the three closed states of I_Ktof_, as shown in **Scheme 5**, where the drug bound states are B1_tof_, B2_tof_, and B3_tof_.
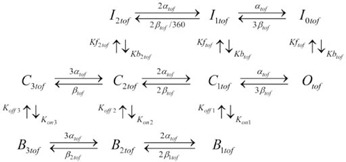
Scheme 5


Experimentally-derived rate constants for I_Ktof_ in mouse ventricular myocytes are different to those for I_Ktof_ in ferret myocytes [12]. The drug association and dissociation rates for drug C were therefore based on those in [8], but transitions between drug bound states were adjusted to match the kinetics of I_Ktof_ in the mouse. The rates for drug association and dissociation are:


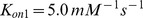



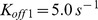



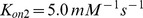



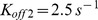



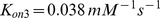



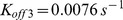



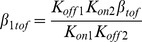



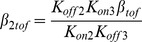


The second hypothetical drug, drug O, was designed to bind only to the open state, since open channel block is probably the most frequently described state-specific mechanism of block. Drug O competitively binds to the open state in the model of I_Ktof_, as shown in Scheme 6. The drug bound state is B_tof_.
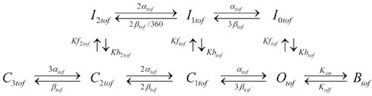
Scheme 6


The rates of association and dissociation for drug O are from [22]. As described in [22], the rates were chosen to mimic the effect of quinidine to cardiac K^+^ channels:


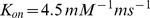



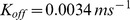


**Figure 1:**
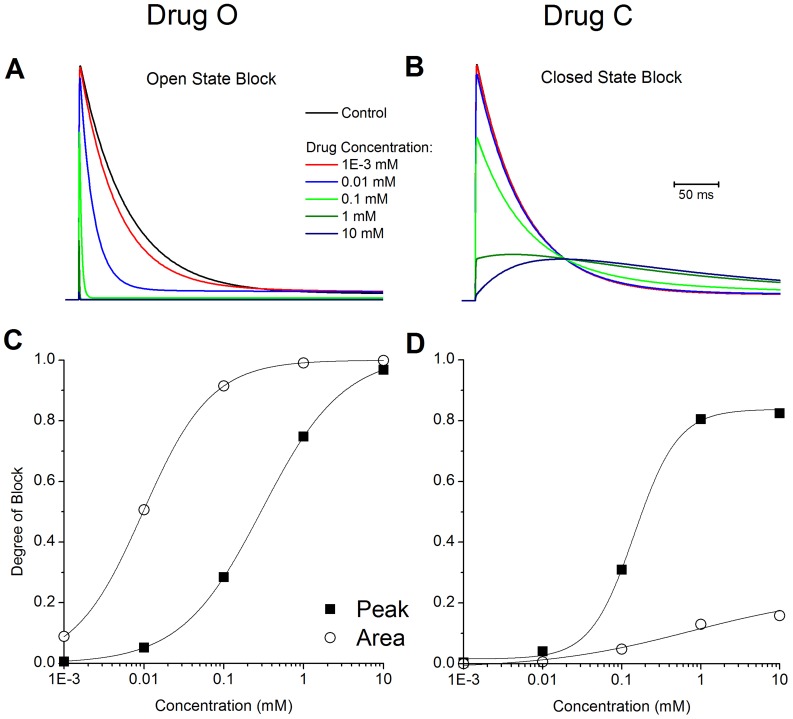
Dose dependent blockade of I_Ktof_. The effect of various concentrations of drug on the action potential for **A:** open state block, and **B:** closed state block. The degree of block was determined by holding at −70 mV, the applying a test pulse to +50 mV for 500 ms. **C:** Open state binding of Drug O. **D:** Closed state binding of Drug C. Change in peak (▪), change in total current flow (○). Solid lines are Boltzmann fits to the data 
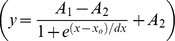
.

We first compared the blocking effect of the two hypothetical drugs on I_Ktof_. We determined dose-response relationships for drug C and O at a test pulse of 50 mV for 500 ms from a holding potential of −70 mV. **Figure 1** shows dose-response curves for both drugs. The effect was measured on the peak current (I_peak_), and on the total current flow (area under the curve). The peak curve was constructed as the normalized reduction in peak I_Ktof_ (1- I_peak-dose_/I_peak-in- control_) as a function of the applied dose of the drug. The area under the curve was constructed as the normalized reduction in the current area (1-Area_dose_/Area_in-control_) as a function of the applied dose of the drug.

For drug C there was no detectable effect on either peak or area current at a concentration of 1 uM. At larger concentrations, the reduction in peak current was much larger than the reduction in current flow (current area) due to the fact that a closed state binding drug (such as 4-AP) slows the activation and time course of current decay of I_Ktof_ [8]. Therefore, although the current peak is reduced, the duration of current flow is increased, resulting in a weaker blocking effect on total current flow. At 10 mM, there is <20% reduction in total current flow, but ∼80% reduction in I_peak_.

For drug O, at a concentration of 1 uM, there is no detectable reduction in I_peak_ but the total current flow is slightly reduced because the open channel blocker accelerates current decay [20], [23]. At a concentration of 1 mM, I_peak_ is ∼80% blocked, and the total current flow is nearly 100% reduced, i.e., there is only a brief rapid transient current with minimal total current flow.

If the drug effect is analyzed only in terms of I_peak_, the IC_50_ value (dose at which the effect is 50% of maximum) of drug O is approximately equal to drug C. Drugs O and C have similar potency on I_Ktof_ only if IC_50_ value is based on I_peak_ at +50 mV.

**Figure 2:**
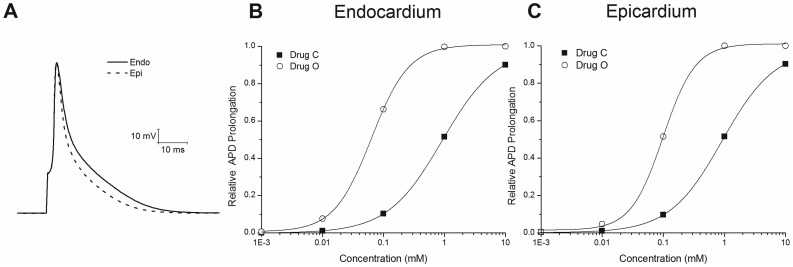
Effect of drugs O and C on APD prolongation. **A:** simulated action potentials of the mouse ventricular model for the epicardial and endocardial cells. Pacing rate was 1 Hz. Relative APD prolongation normalized to the maximum prolongation with I_Ktof_ completely blocked was determined at various drug concentrations for drug O (○) and drug C (▪) on **B:** endocardium and **C:** Epicardium. Pacing rate was 1 Hz.

**Figure 2A** shows drug free traces from epicardial and endocardial cells. Next, we determined the effect of drugs O and C binding to I_Ktof_ on prolonging the ventricular APD. Action potential duration was measured as the interval between (dV/dt)max on the upstroke and 50% of repolarization (APD50), The myocyte was paced at 1 Hz with various concentrations of drug, or drug free. The prolongation in APD50 was normalized to the maximum prolongation achieved with I_Ktof_ completely blocked. This test was conducted for both the endocardial and epicardial cell models.

**Figure 2** **B and C** shows the effect of drugs O and C on APD for various drug concentrations. The relative degree of APD prolongation is determined relative to the prolongation obtained with 100% block of I_Ktof_. In endocardial cells, 50% of maximal APD prolongation is obtained with 0.96 mM drug C and 0.06 mM drug O. In epicardial cells, 50% of maximal prolongation is obtained at 0.97 mM for drug C and 0.10 mM for drug O. Therefore, in terms of APD50 prolongation, 1 mM drug C can be considered as having approximately the same potency as 0.1 mM drug O at a pacing rate of 1 Hz.

**Figure 3:**
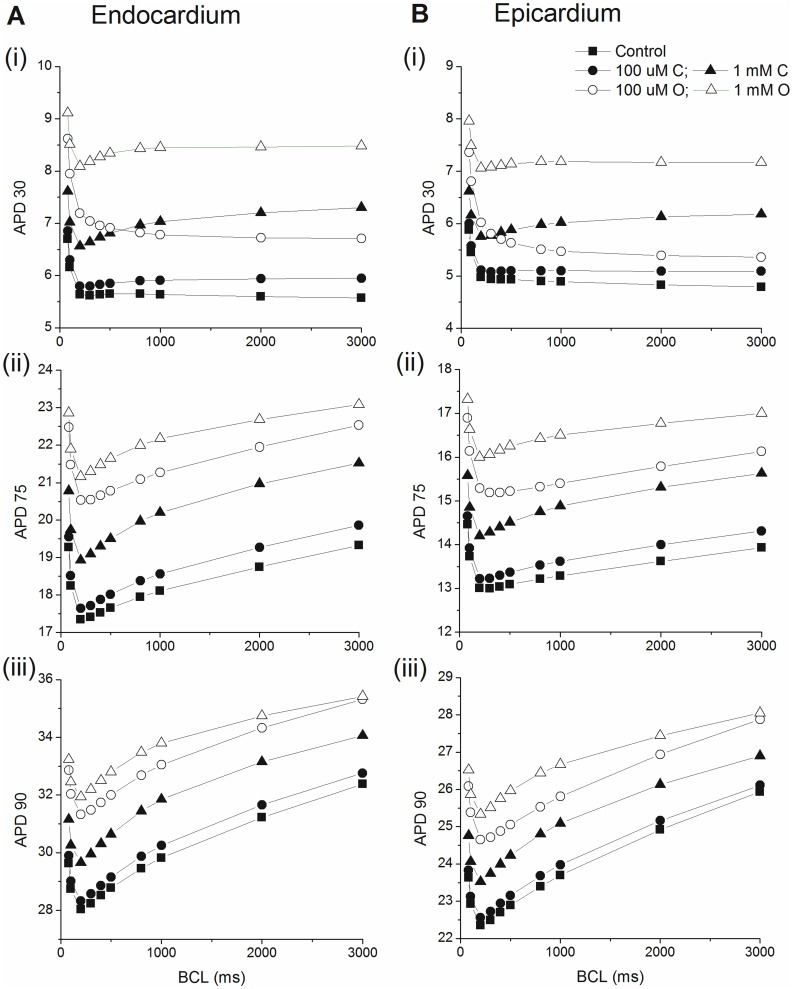
APD-BCL relationships. **A:** endocardium and **B:** epicardium. (i) APD 30; (ii) APD75; (iii) APD90. Control in the absence of drug (▪), 0.1 mM drug C (•), 1 mM drug C (▴), 0.1 mM drug O (○), 1 mM drug O (Δ).

Based on these results, we chose 0.1 (drug O) and 1 mM (drug C) as the test doses to characterize the steady-state rate dependence and the restitution kinetics of the mouse ventricular myocytes. First, we calculated the steady state APD-cycle length relationships of the endocardial and epicardial cell models. Simulations were conducted at the following basic cycle lengths: 80, 100. 200, 300, 400, 500, 800, 1000, 2000, and 3000 ms. The stimulus for all simulations was an 0.5 ms pulse of 60 pA/pF. For each BCL, we paced for 1000 beats and used the last beat to calculate the APD30, APD75, and APD90 values (the interval between (dV/dt)max on the upstroke and 30%, 75% and 90% repolarization respectively). Simulations were conducted in control and with 0.1 and 1 mM of drugs O and C. The resulting APD-BCL relationships are shown in **Fig 3**.

Epicardial APD is shorter than the endocardial APD, due to the increased magnitude of several outward K^+^ currents. Under all conditions, for BCL ≥200 ms, both the epicardial and endocardial cells show an increase in APD90 and APD75 when BCL increases. For BCL<200 ms, APD90 and APD75 increase with shortening BCL (as observed experimentally [24]), therefore all APD-BCL curves show a V-shape with the turning point at ∼200 ms. For all BCLs tested, APD90 and APD75 were only slightly prolonged by 0.1 mM drug C. 1 mM drug C and drug O at both doses prolong APD90 and APD75 significantly, with the strongest effect caused by 1 mM drug O and the weakest effect by 1 mM drug C. 0.1 mM drug O always has weaker effect in prolonging APD than 1 mM drug C. However, for APD90, the effect of 0.1 mM drug O tends to approach that of 1 mM drug C with increasing BCL, reaching almost the same level at BCL = 3000 ms.

**Figure 4:**
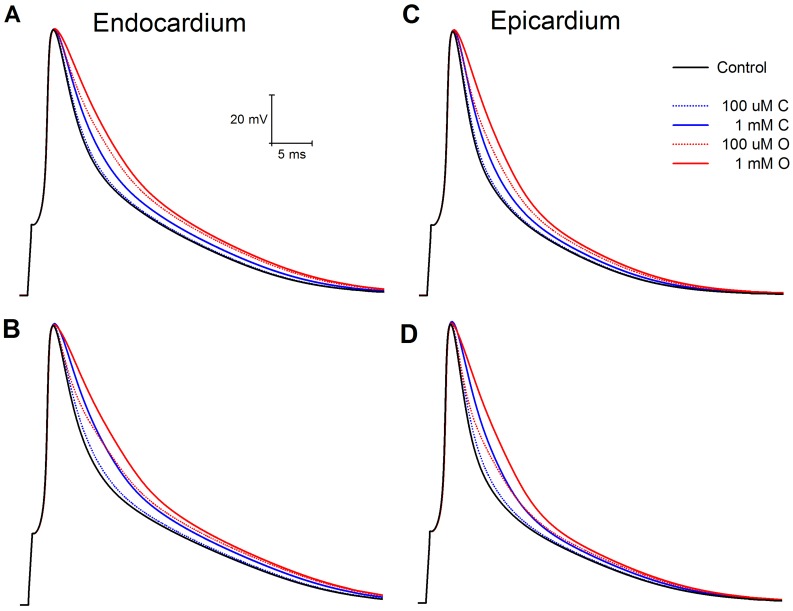
Comparison of action potentials from endocardium and epicardium at fast and slow pacing rates. Endocardium: **A:** 200 ms **B:** 2s. Epicardium: **C:** 200 ms **D:** 2 s. Control is solid black line. Drug O is red, drug C blue.

An interesting phenomenon happens at APD30. First, in the presence of 0.1 mM drug O, both the endocardial and epicardial model show increasing APD30 with shortening BCL for all BCLs tested, in contrast to the V-shaped curve obtained in control and other drug conditions. Second, because of the consistently decreasing APD30 with increasing BCL, the curve for 0.1 mM drug O crosses over the curve for 1 mM drug C at a point such that for BCLs longer than that point (∼300 ms for epicardium and 600 ms for endocardium), 1 mM drug C has a stronger effect in prolonging APD30 than 0.1 mM drug O, which does not happen for APD75 and APD90. To illustrate this, **Figure 4** shows APs at 200 ms and 2s for endocardial and epicardial cells. At 200 ms, AP repolarization in the presence of 0.1 mM drug O is always more rapid (i.e., shorter APD) than for 1 mM drug C. However, at 2s, initial early AP repolarization with 0.1 mM drug O is more rapid, then subsequently less rapid than for 1 mM drug C later in repolarization. Therefore, 1 mM drug C has a longer APD30 but shorter APD75 and APD90 than 0.1 mM drug O.

**Figure 5:**
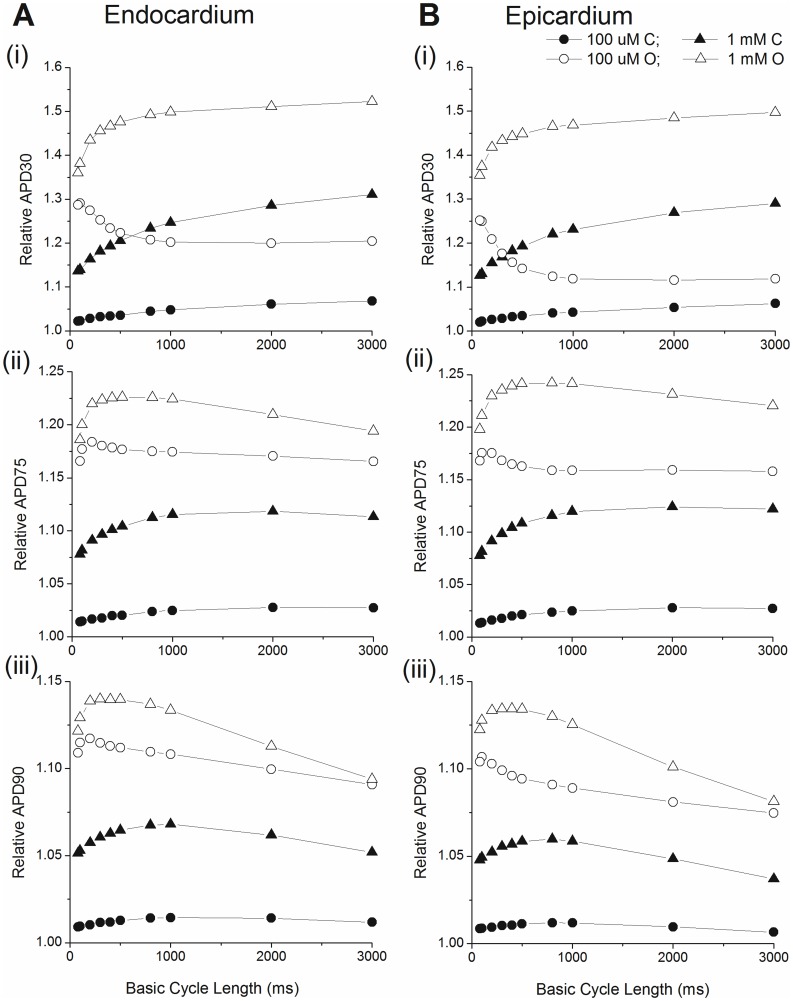
Relative APD-BCL relationships. **A:** endocardium and **B:** epicardium. (i) APD 30; (ii) APD75; (iii) APD90. Simulations are with 0.1 mM drug C (•), 1 mM drug C (▴), 0.1 mM drug O (○), 1 mM drug O (Δ). All APDs are normalized for control in the absence of drug.

To further compare the different effects of drug C and O on the APD-BCL relationships, we normalized the APDs at each BCL in the presence of drugs to their corresponding APD at control, as shown in **Figure 5**.

Based on this we conclude: 1) the effect in prolonging APD caused by both drugs are BCL dependent since relative APD changes with varying BCL. 2) The effect in prolonging APD caused by both drugs differed dramatically between early, middle, and late repolarization. For APD30, both doses of drug C and 1 mM drug O all prolonged APD30 more with increasing BCL (reverse use dependency). But 0.1 mM drug O showed the opposite, prolonging APD30 less with increasing BCL. The relative APD30 curve for 1 mM drug C crosses that for 0.1 mM drug O at a certain point (just as in Figure 5), indicating stronger effect in prolonging APD by 1 mM drug C than 0.1 mM drug O for slower pacing rate. For APD75 and APD90, both doses of drug O showed decreasing relative APD with increasing BCL for BCL>200 ms, indicating weaker drug effect with slower pacing, a property of use-dependency. Drug C, however, showed reverse use dependency for APD75 in the whole range of BCLs tested and for APD90 in the fast pacing range (BCL shorter than about 1s). For APD90 in the slow pacing range (BCL longer than 1s), drug C showed use dependency.

**Figure 6:**
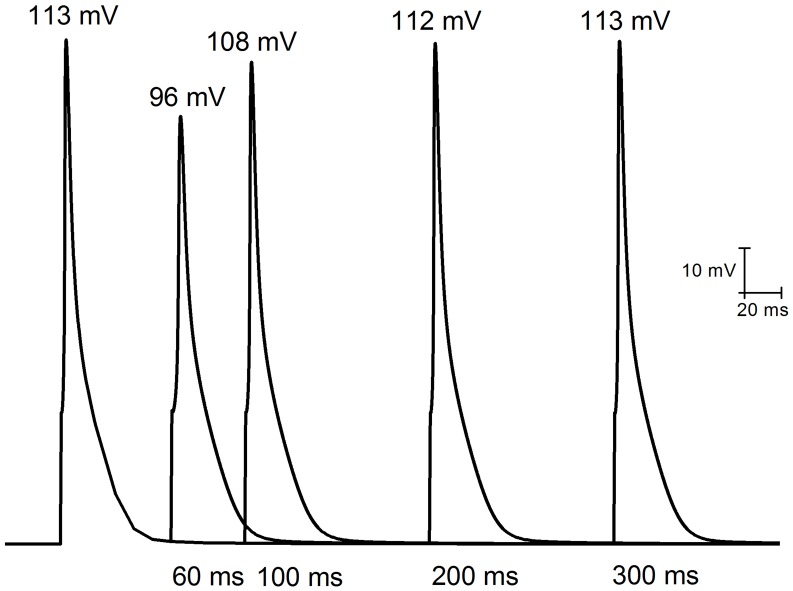
Example of APs recorded with different S1–S2 intervals. The first AP (control) represents the last beat of the pacing train at a cycle length of 1s (S1) on endocardial cells. APs are shown for S1–S2 intervals of 60, 100, 200, and 300 ms. Peak amplitudes are shown above the AP. APD30 is 4.89 ms, 8.17 ms, 6.11 ms, 5.58 ms, and 5.56 ms; APD75 is 18.11 ms, 22.07 ms, 18.65 ms, 17.81 ms, and 17.81 ms; and APD90 is 29.82 ms, 32.73 ms, 30.13 ms, 29.54 ms, and 29.47 ms for control, 60, 100, 200, and 300 ms S1–S2 intervals respectively.

**Figure 7:**
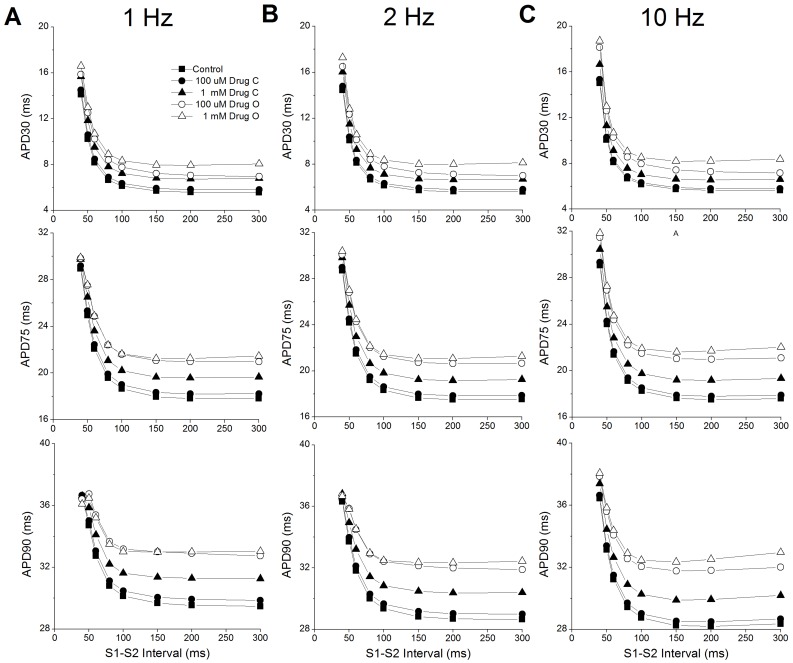
Restitution curves for endocardial cells. **A:** 1 Hz pacing; **B:** 2 Hz pacing; **C:** 10 Hz pacing. Top: APD30; Middle: APD75; Bottom: APD30. Control in the absence of drug (▪), 0.1 mM drug C (•), 1 mM drug C (▴), 0.1 mM drug O (○), 1 mM drug O (Δ).

**Figure 8:**
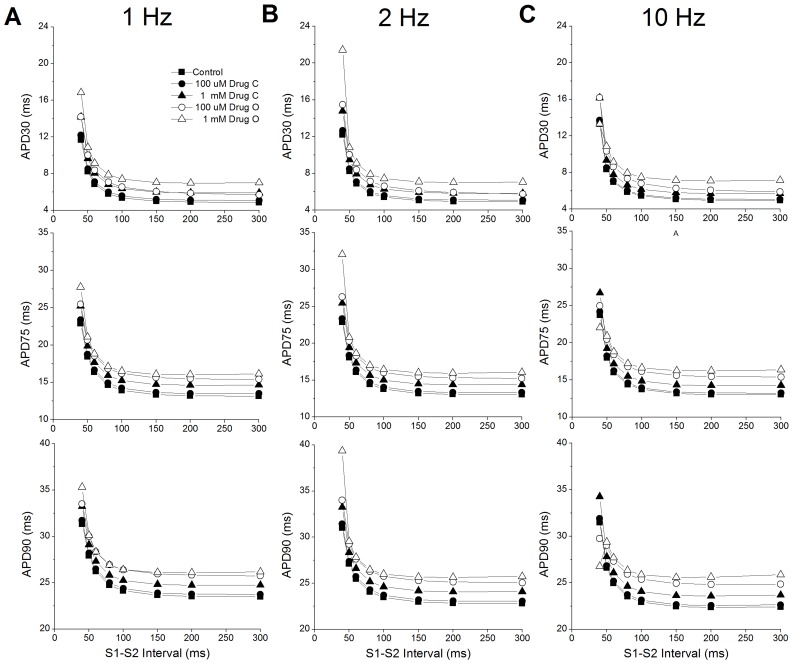
Restitution curves for epicardial cells. **A:** 1 Hz pacing; **B:** 2 Hz pacing; **C:** 10 Hz pacing. Top: APD30; Middle: APD75; Bottom: APD30. Control in the absence of drug (▪), 0.1 mM drug C (•), 1 mM drug C (▴), 0.1 mM drug O (○), 1 mM drug O (Δ).

We then tested restitution at various drug concentrations. Each model was paced with a train of 1000 beats (S1) at three rates: 1s, 500 ms and 100 ms, followed by a single S2 beat with a shorter S1–S2 interval. The S1–S2 interval varied from 40 ms (the smallest duration which can generate a reasonable AP) to 300 ms. **Figure 6** shows the last beat of the train of pacing (S1 = 1 Hz) with superimposed premature responses at successively longer S1–S2 intervals in control conditions. For an S1–S2 interval of 60 ms, the AP amplitude of the S2 beat is smaller than that of S1. With increasing S1–S2 intervals, the amplitude of the S2 APs becomes larger and gradually recovers to the steady state magnitude. This agrees very well with experimental observations [25]. However, our simulation results do not show a shortened APD90 at short S1–S2 levels. The S1–S2 restitution curves for endocardial and epicardial cell models are shown in **Figures 7** and **8** respectively.

With few exceptions, the restitution curves show a negative initial slope, i.e., APD shortens with increasing S1–S2 intervals. At longer S1–S2 intervals, APD reaches steady state or shows slight APD prolongation with increasing S1–S2 interval. At all pacing rates, restitution with 0.1 mM drug C is similar to control, with only modest APD prolongation. The shape of the restitution under 1mM drug C resembles that of control, although with larger prolongation in APD than 0.1 mM drug C. Interestingly, restitution with 0.1 mM drug O is closer to the restitution with 1 mM drug C for APD30, but closer to the restitution with 1 mM drug O for APD75 and APD90.

The exceptions to the negative initial restitution slope are: APD90 in endocardial and epicardial cells paced at 1 Hz, with an S1–S2 interval under 50 ms which has a ‘hook’ in the beginning of the restitution curves with 0.1 and 1 mM drug O. At an S1–S2 interval of 40 ms, all APDs with 1mM drug O are shorter than those under control and other conditions and APD90 and APD75 with 0.1 mM drug O are shorter than those with 1 mM drug C.

**Figure 9:**
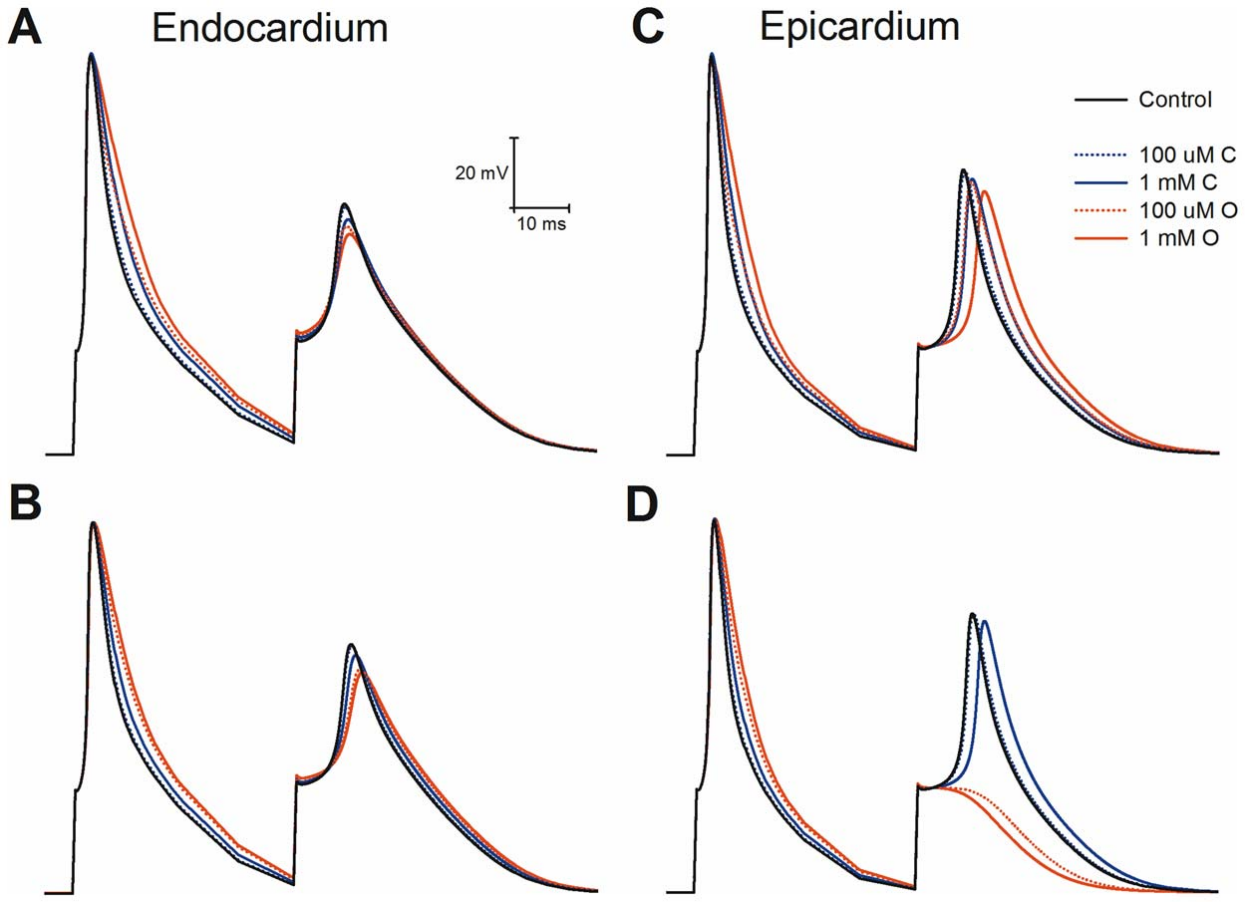
Comparison of action potentials at with S1S2 interval at 40 ms. Endocardium was stimulated at **A:** 1 Hz **B:** 10 Hz. Epicardium was stimulated at **C:** 1 Hz **D:** 10 Hz. Control, in the absence of drug is indicated by solid black line. Drug C is shown in blue, and drug O in red.

The AP traces for the final S1 and S2 beat are shown in **Figure 9** for the S1–S2 interval of 40 ms at 1 and 10 Hz pacing. The shape of the S2 AP varies greatly. For endocardial cell at 1s pacing rate, the drugs suppress the peak AP depolarization but have no effect on repolarization or APD. For endocardial cell at 10 Hz pacing, the peak depolarization is suppressed and also delayed, followed by delayed repolarization. For epicardial cell at 1 Hz pacing, the AP peaks depolarization is reduced and delayed. At 10 Hz pacing, drug C delayed and reduced the AP peak, whereas drug O abolished the second action potential.

## Discussion

The mouse has become an increasingly well used model to study the molecular, cellular and systemic mechanisms underlying cardiac arrhythmias. Due to the ease with which genetic manipulations can be applied, the mouse has proved to be a powerful tool in providing considerable mechanistic information concerning ion channel gating, and its modification by either channel mutation or pharmacological intervention. In order to take advantage of the available channel level data and to use them to predict how the kinetic consequences of many transgenic or pharmacological modifications on channel properties lead to changes in AP behavior, comprehensive mathematical models of mouse myocytes have to be constructed. Our new mouse ventricular myocyte AP models developed here use Markov formulation for all major ion currents, including the fast sodium current I_Na_, the L-type calcium current I_CaL_, the rapid delayed rectifier potassium current I_Kr_, the rapidly inactivating transient outward current I_Ktof_, the slow-inactivating transient outward current *I*_Ktos_, the ultra-rapidly activating delayed-rectifier current I_Kur_, and the slow delayed-rectifier current I_Ks_. For the four channels that have been newly updated to Markov models (I_Ktof_, I_Ktos_, I_Kur_, and I_Ks_), the new models either reproduced the properties of the old models, if the old model agreed with available data, or produced better results than the old ones, if the old model did not agree with data. The new AP models reproduce the APs of two types of cardiac myocytes: enodcardial and epicardial cells. These new models can be used as a platform for the integration and interpretation of channel gating data, and for the exploration of the mechanisms of cardiac arrhythmia. As shown in this paper, the new models were used to successfully study the different effects of the two hypothetical drugs on changing AP behavior.

Conventionally, potency of pharmaceutical compounds is characterized by measuring their IC_50_ values (the dose at which the target current is half blocked in peak value under a certain protocol). However, IC_50_ values cannot fully describe drug potency as they only apply to one specific condition under which they are measured. When different measurements are made, different IC_50_ values will be obtained and therefore they are inadequate indicators of drug potency. As shown in **Figure 1**, if peak value was considered, drug C (closed state binding) seemed to have similar IC_50_ as drug O. However, if current area was measured, drug O (IC_50_ = 10 uM) showed much stronger blocking effect than drug C (maximum block less than 20%). Furthermore, when the drug blocked channels were incorporated into the whole cell model and APD was measured, the IC_50_ values changed again. At a pacing rate of 1 Hz and when APD50 was measured, IC_50_ was 1 mM for drug C and about 0.1 mM for drug C (**Figure 2**). When different rates and APD at different repolarization stage were used, the dose dependence curves were to change again. Therefore, a comprehensive description of drug effects on AP requires the measurement of the steady state rate dependence and restitution in the presence of the drug.

Drugs which alter repolarization are generally referred to as class III anti-arrhythmic drugs. The general principle underlying the action of these compounds is that by increasing repolarization time, refractorieness can be increased [26]. By increasing the amount of refractory tissue, reentrant arrhythmias could be suppressed [27]. With the discovery and identification of several forms of long QT syndrome occurring from reductions in potassium currents [3], and the unfortunate outcomes of the CAST I and CAST II trials [28], [29], this approach to suppressing arrhythmias in ventricle has fallen out of favor. However, it remains a viable approach in suppressing arrhythmias in atrium [30]–[32]. The currents underlying the mouse action potential more closely align with human atrium than the human ventricle as repolarization in both human atria and mouse ventricle have strong components of a transient outward current and a Kv1.5 mediated component which dominate repolarization [33], [34]. At very short cycle lengths the mouse APD, when measured at positive potentials, can actually increase [24] due to incomplete recovery from inactivation of the transient outward current. This anomalous increase in the very short mouse APD may be similar in mechanism to rate dependent changes in the “notch” or Phase 1 portion of human ventricular repolarization.

What constitutes an optimal channel to block or an optimal change in action potential for arrhythmia suppression is still ill defined. In-silico methods will undoubtedly play an important role in developing better criteria for drug development [35]. This type of analysis will include higher levels of integration beyond the action potential, such as propagated behavior which may show additional levels of complexity not predicted solely from cellular action potentials [36]. Our study demonstrates that the restitution properties of the action potential can be strongly dependent upon mechanism and kinetics of block and that consideration of state-dependent binding is essential.

## Materials and Methods

### Mathematical Model

The derivation of our original mouse model is detailed in Bondarenko *et al.* [7]. The model has 40 ordinary differential equations solved by a fourth-order Runge-Kutta method, with variable step size and implemented in Microsoft Visual C++2008. Computations were performed on a Dell Precision T7500 with 2 Intel Xeon CPU E5520. Numerical accuracy was confirmed by demonstrating insensitivity to step size. Steady-state initial conditions were obtained by running the model until changes in all variables did not exceed 0.01%.The whole cell model membrane potential, *V*, was calculated using the following equation:


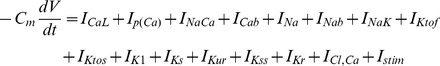


The detailed model equations for each component current are given in Bondarenko et al. [7]. The four currents for which we derived new Markov Model representations are: the rapidly inactivating transient outward K^+^ current I_Ktof_; the slowly-inactivating transient outward K^+^ current I_Ktos_; the slow delayed-rectifier K^+^ current I_Ks_; and the ultrarapidly activating delayed rectifier K^+^ current I_Kur_.

### Parameter Determination and Optimization

Model parameters for the Markov models of two transient outward currents were adapted from the original HH formulations of Bondarenko *et al* [7]. In the case of I_Ktof_ the core activation process used the HH expansion of existing parameters and inactivation was coupled using the process described in Campbell *et al* for ferret ventricular myocytes [12]. The simulated recovery currents were adjusted manually to match the original model. Closed state drug binding kinetics were taken directly for 4-Aminopyridine from Campbell *et al* [8]. Drug binding kinetics for open channel binding were taken directly from Liu and Rasmusson [22]. The Kv1.4 mediated I_Ktos_ and the coupled inactivation gating structure was taken from the recent analysis of Kv1.4 heterologously expressed potassium channels [14], final parameter values were obtained by manual variation and matching steady state inactivation, inactivation rate and recovery rate to simulated HH values. As no data exist quantifying the delayed rectifier current, I_KS_, the values and structure for this gating model were taken directly from [16]. The model for the I_Kur_ component was adapted by expanding the HH formulation for activation and coupling a single inactivated state to the open state, the forward rate was determined algebraically from the time constant of inactivation and recovery reported by Xu et al. [11], [37]. Simulation protocols are as detailed in the text.

## Supporting Information

Figure S1 **The rapidly inactivating transient outward K^+^ current I_Ktof_.** Simulated traces were obtained from a 500 ms P1 pulse to between −100 and +50 mV (20 mV steps) from the holding potential of −80 mV followed by a 500 ms P2 pulse to +50 m V. **A:** Traces from the new Markov model. **B:** Traces from the Bondarenko model with an HH formalism. **C:** Peak I_Ktof_ in P1 vs. P1 voltage from the Bondarenko model (▪) and the Markov model (○), with 10 mV steps. **D:** Steady state inactivation. Peak current in the P2 pulse is plotted against P1 voltage from simulations from the Bondarenko model (▪) and the Markov model (○). The Markov simulated steady-state inactivation curve is shifted slightly negative compared to the Bondarenko simulation. This shift was introduced to compensate for the “surface charge” effects of divalent ions used to block overlapping Ca^2+^ currents [38] during the experiments. This correcting shift had been accounted for in the activation process for the Bondarenko model, but had not been incorporated into the inactivation gating variable in the old HH formulation. The result is a much better simulation of the substantial closed state inactivation observed for Kv4 channels [39], [40].
(TIF)

Figure S2 **The slow-inactivating transient outward K+ current I_Ktos_.** Simulated traces were obtained from a 2 s pulse to between −50 and +50 mV (in 20 mV increments) from the holding potential of −100 mV. **A:** Traces from the Markov model. **B:** Traces from the Bondarenko model. **C:** Peak I_Ktos_-voltage relationships from the Bondarenko model (▪) and the Markov model (○). **D:** Steady state inactivation relationships from the Bondarenko model (▪) and the Markov model (○). The rate of inactivation in the Markov model becomes voltage insensitive at positive potentials which is more consistent with experimental data and observed mechanisms [14], [41].
(TIF)

Figure S3 **The ultra-rapidly activating delayed rectifier K^+^ current I_Kur_.** Simulated traces were obtained from a 5 s pulse to between −50 and +50 mV (in 20-mV increments) from the holding potential of −70 mV. **A:** Traces from the Markov model. **B:** Traces from the Bondarenko model. **C**: Peak I_Kur_-voltage relationships from the Bondarenko model (▪) and the Markov model (○). **D:** Steady state inactivation relationships from the Bondarenko model (▪) and the Markov model (○). Current is measured at the end of a 2.5 s test pulse at +30 mV preceded by a 5 s conditioning pulse to various potentials between −110 and −20 mV for 5s, and a 100 ms inactivating prepulse to −40 mV. In the new Markov model, inactivation is incomplete at depolarized voltages, which is in better agreement with experimental data on cloned Kv1.5 channels [42], [43].
(TIF)

Text S1 **Model Equations.**
(DOC)
