# Supplementary figures and images for: Markov Models of Use-Dependence and Reverse Use-Dependence during the Mouse Cardiac Action Potential
Source: PLoS One. 2012 Aug 6;7(8):e42295. doi: 10.1371/journal.pone.0042295 (PMC3412869; doi:10.1371/journal.pone.0042295)

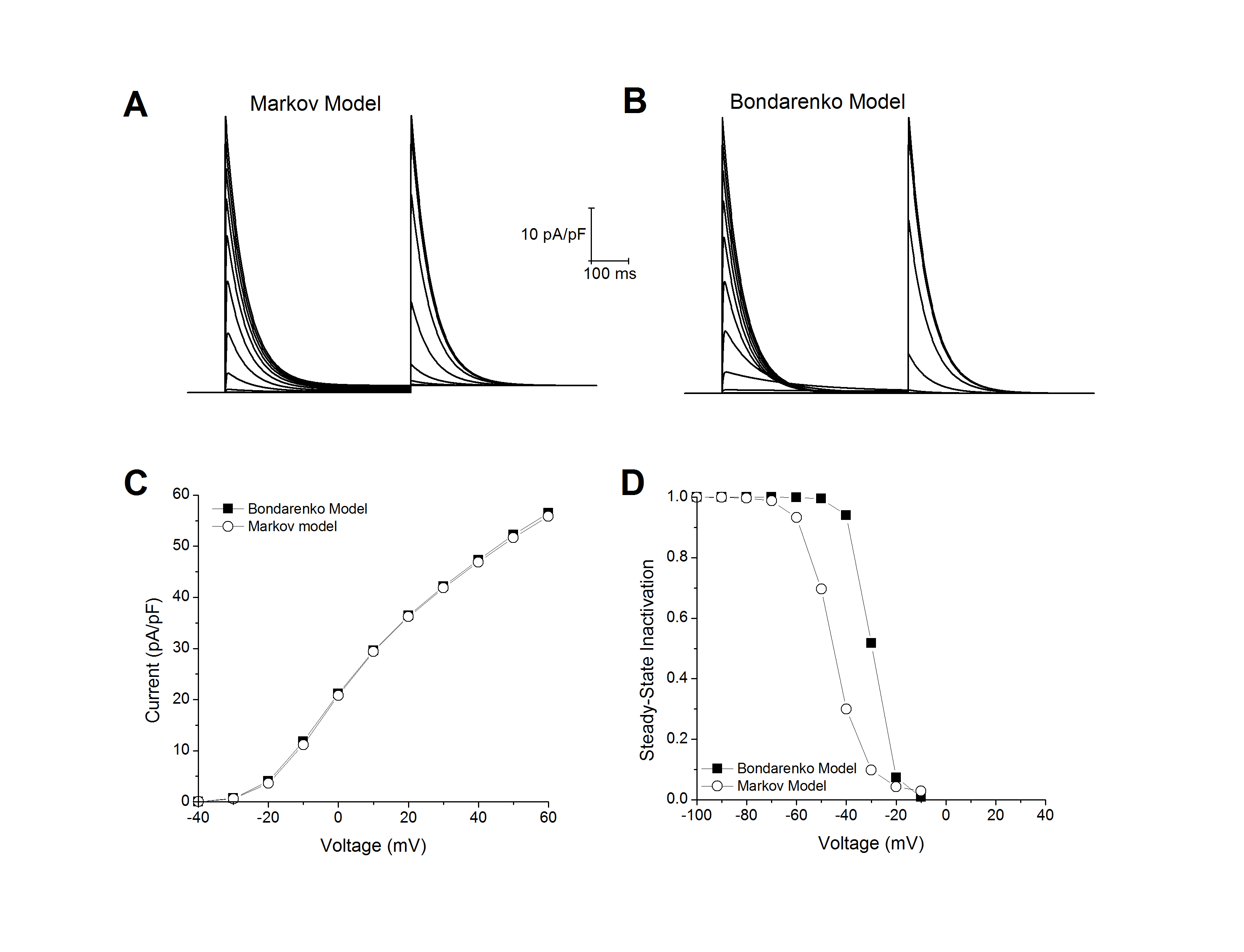

Supplement: Figure S1 — The rapidly inactivating transient outward K+ current IKtof. Simulated traces were obtained from a 500 ms P1 pulse to between −100 and +50 mV (20 mV steps) from the holding potential of −80 mV followed by a 500 ms P2 pulse to +50 m V. A: Traces from the new Markov model. B: Traces from the Bondarenko model with an HH formalism. C: Peak IKtof in P1 vs. P1 voltage from the Bondarenko model (▪) and the Markov model (○), with 10 mV steps. D: Steady state inactivation. Peak current in the P2 pulse is plotted against P1 voltage from simulations from the Bondarenko model (▪) and the Markov model (○). The Markov simulated steady-state inactivation curve is shifted slightly negative compared to the Bondarenko simulation. This shift was introduced to compensate for the “surface charge” effects of divalent ions used to block overlapping Ca2+ currents [38] during the experiments. This correcting shift had been accounted for in the activation process for the Bondarenko model, but had not been incorporated into the inactivation gating variable in the old HH formulation. The result is a much better simulation of the substantial closed state inactivation observed for Kv4 channels [39], [40]. (TIF) [file pone.0042295.s001.tif]

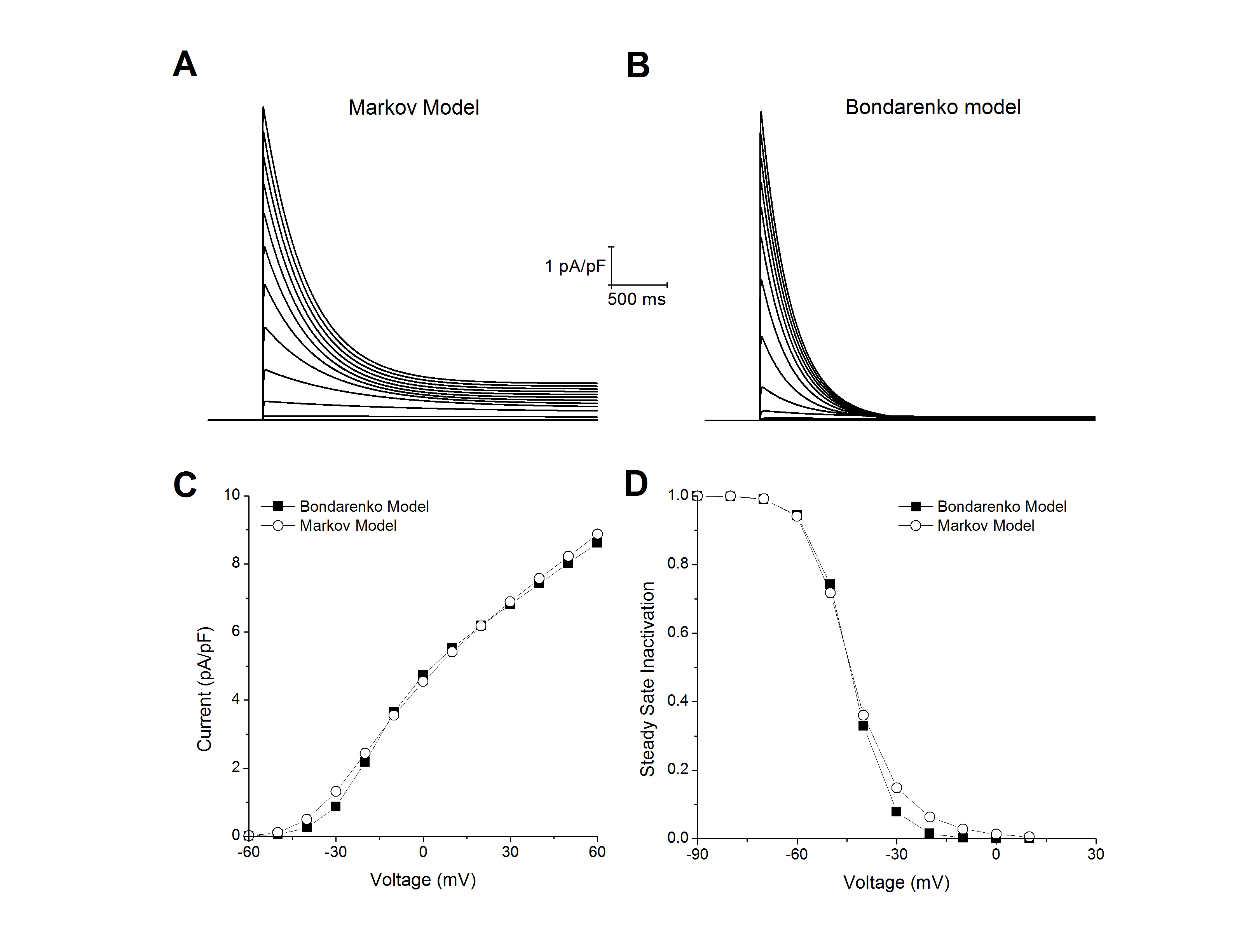

Supplement: Figure S2 — The slow-inactivating transient outward K+ current IKtos. Simulated traces were obtained from a 2 s pulse to between −50 and +50 mV (in 20 mV increments) from the holding potential of −100 mV. A: Traces from the Markov model. B: Traces from the Bondarenko model. C: Peak IKtos-voltage relationships from the Bondarenko model (▪) and the Markov model (○). D: Steady state inactivation relationships from the Bondarenko model (▪) and the Markov model (○). The rate of inactivation in the Markov model becomes voltage insensitive at positive potentials which is more consistent with experimental data and observed mechanisms [14], [41]. (TIF) [file pone.0042295.s002.tif]

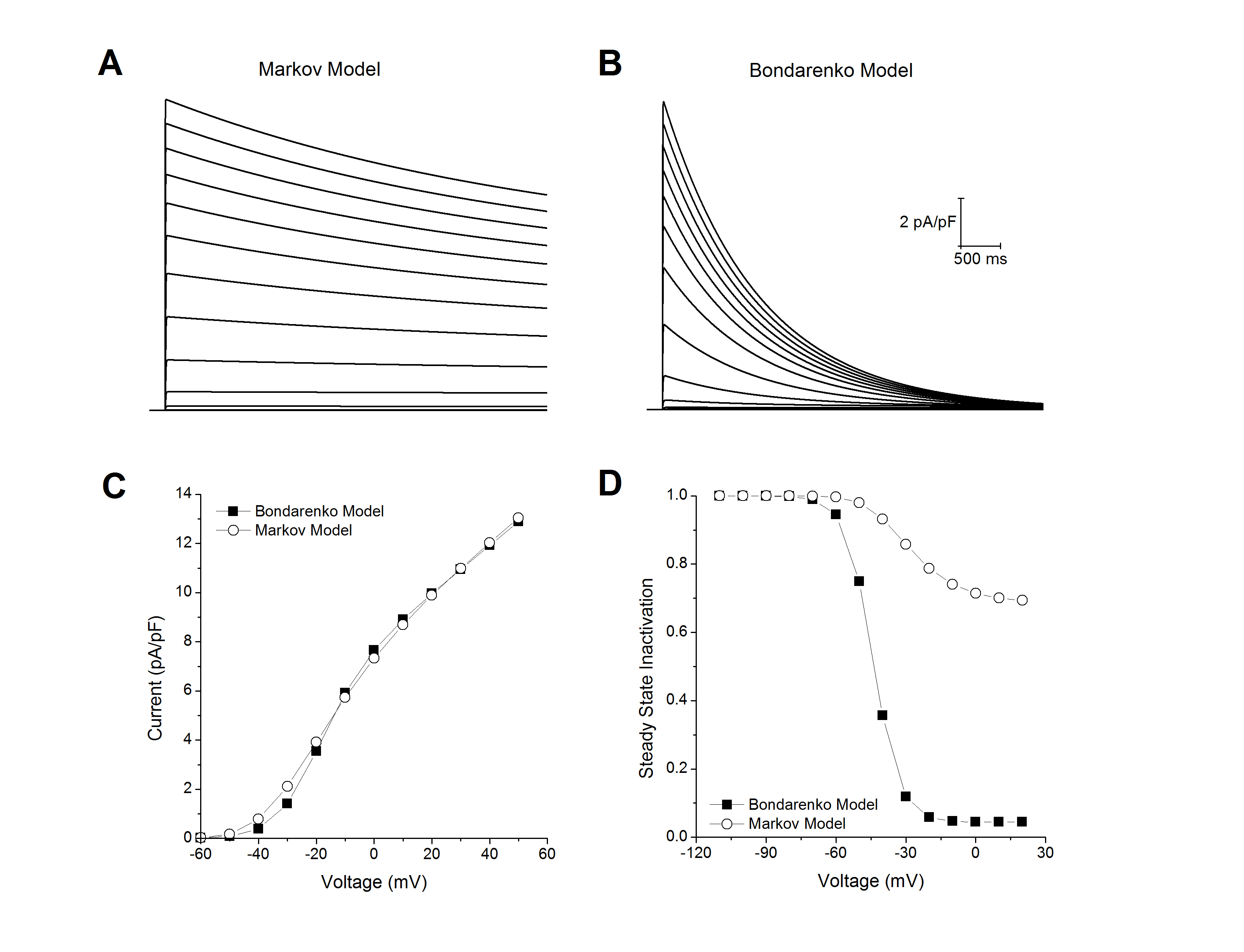

Supplement: Figure S3 — The ultra-rapidly activating delayed rectifier K+ current IKur. Simulated traces were obtained from a 5 s pulse to between −50 and +50 mV (in 20-mV increments) from the holding potential of −70 mV. A: Traces from the Markov model. B: Traces from the Bondarenko model. C: Peak IKur-voltage relationships from the Bondarenko model (▪) and the Markov model (○). D: Steady state inactivation relationships from the Bondarenko model (▪) and the Markov model (○). Current is measured at the end of a 2.5 s test pulse at +30 mV preceded by a 5 s conditioning pulse to various potentials between −110 and −20 mV for 5s, and a 100 ms inactivating prepulse to −40 mV. In the new Markov model, inactivation is incomplete at depolarized voltages, which is in better agreement with experimental data on cloned Kv1.5 channels [42], [43]. (TIF) [file pone.0042295.s003.tif]
