# Supplementary material for: Markov Models of Use-Dependence and Reverse Use-Dependence during the Mouse Cardiac Action Potential
Source: PLoS One. 2012 Aug 6;7(8):e42295. doi: 10.1371/journal.pone.0042295 (PMC3412869; doi:10.1371/journal.pone.0042295)
Supplement: Text S1 — Model Equations. (DOC) [file pone.0042295.s004.doc]

**Model Equations**

Rapidly inactivating transient outward K^+^ current I_Kto,f_

$$\text{C}_{\text{3tof}}=1-C_{2tof}-C{}_{1tof}-I_{2tof}-I_{1tof}-I_{0tof}-O_{tof}$$

$$\frac{\text{dC}_{\text{2tof}}}{\text{dt}}=3\alpha_{tof}C_{3tof}-\beta_{tof}C_{2tof}+2\beta_{tof}C{}_{1tof}-2\alpha_{tof}C_{2tof}+K_{b2tof}I_{2tof}-K_{f2tof}C_{2tof}$$

$$\frac{\text{dC}_{\text{1tof}}}{\text{dt}}=2\alpha_{tof}C_{2tof}-2\beta_{tof}C_{1tof}+3\beta_{tof}O{}_{tof}-\alpha_{tof}C_{1tof}+K_{btof}I_{1tof}-K_{ftof}C_{1tof}$$

$$\frac{\text{dO}_{\text{tof}}}{\text{dt}}=\alpha_{tof}C_{1tof}-3\beta_{tof}O_{tof}+K_{btof}I_{0tof}-K_{ftof}O_{tof}$$

$$\frac{\text{dI}_{\text{0tof}}}{\text{dt}}=\alpha_{tof}I_{1tof}-3\beta_{tof}I_{0tof}+K_{ftof}O_{tof}-K_{btof}I_{0tof}$$

$$\frac{\text{dI}_{\text{1tof}}}{\text{dt}}=2\alpha_{tof}I_{2tof}-2\beta_{tof}I_{1tof}/360+3\beta_{tof}I{}_{0tof}-\alpha_{tof}I_{1tof}+K_{ftof}C_{1tof}-K_{btof}I_{1tof}$$

$$\frac{\text{dI}_{\text{2tof}}}{\text{dt}}=2\beta_{tof}I_{1tof}/360-2\alpha_{tof}I_{2tof}+K_{f2tof}C_{2tof}-K_{b2tof}I_{2tof}$$

$$\alpha_{tof}=0.18064e^{0.03577(V+30.0)}$$

$$\beta_{tof}=0.3956e^{-0.06237(V+30.0)}$$

$$\text{K}_{\text{ftof}}=0.0183ms^{-1}$$

$$\text{K}_{\text{btof}}=K_{ftof}/40$$

$$\text{K}_{\text{f2tof}}=0.0183ms^{-1}$$

$$\text{K}_{\text{b2tof}}=360K_{f2tof}K_{btof}/K_{ftof}$$

Slow-inactivating transient outward K^+^ current I_Kto,s_

$$\text{C}_{\text{1tos}}=1-C_{2tos}-C{}_{3tos}-C{}_{4tos}-I_{C2tos}-I_{C1tos}-I_{Ntos}-I_{tos}-O_{tos}$$

$$\frac{\text{dC}_{\text{2tos}}}{\text{dt}}=4\alpha_{tos}C_{1tos}-\beta_{tos}C_{2tos}+2\beta_{tos}C{}_{3tos}-3\alpha_{tos}C_{2tos}$$

$$\frac{\text{dC}_{\text{3tos}}}{\text{dt}}=3\alpha_{tos}C_{2tos}-2\beta_{tos}C_{3tos}+3\beta_{tos}C{}_{4tos}-2\alpha_{tos}C_{3tos}$$

$$\frac{\text{dC}_{\text{4tos}}}{\text{dt}}=2\alpha_{tos}C_{3tos}-3\beta_{tos}C_{4tos}+4\beta_{tos}O{}_{tos}-\alpha_{tos}C_{4tos}+K_{bctos}I_{C1tos}-K_{fctos}C_{4tos}$$

$$\frac{\text{dO}_{\text{tos}}}{\text{dt}}=\alpha_{tos}C_{4tos}-4\beta_{tos}O_{tos}+K_{bctos}I_{C2tos}-K_{fctos}O_{tos}+K_{bntos}I_{Ntos}-K_{fntos}O_{tos}$$

$$\frac{\text{dI}_{\text{C1tos}}}{\text{dt}}=K_{fctos}C_{4tos}-K_{bctos}I_{C1tos}+4\beta_{tos}I_{C2tos}-\alpha_{tos}I_{C1tos}$$

$$\frac{\text{dI}_{\text{C2tos}}}{\text{dt}}=K_{fctos}O_{tos}-K_{bctos}I_{C2tos}+\alpha_{tos}I_{C1tos}-4\beta_{tos}I_{C2tos}+K_{bntos}I_{tos}-K_{fntos}I_{C2tos}$$

$$\frac{\text{dI}_{\text{Ntos}}}{\text{dt}}=K_{bctos}I_{tos}-K_{fctos}I_{Ntos}+K_{fntos}O_{tos}-K_{bntos}I_{Ntos}$$

$$\frac{\text{dI}_{\text{tos}}}{\text{dt}}=K_{fctos}I_{Ntos}-K_{bctos}I_{tos}+K_{fntos}I_{C2tos}-K_{bntos}I_{tos}$$

$$\alpha_{tos}=e^{(V-48.4)/49.2}$$

$$\beta_{tos}=e^{(V-48.4)/49.2}\frac{e^{-(V+48.4)/13.6}}{1+0.023e^{-(V+48.4)/13.6}}$$

$$\text{K}_{\text{fntos}}=0.0023ms^{-1}$$

$$\text{K}_{\text{bntos}}=0.0003ms^{-1}$$

$$\text{K}_{\text{fctos}}=0.0023ms^{-1}$$

$$\text{K}_{\text{bctos}}=0.3ms^{-1}$$

Slow delayed-rectifier K^+^ current I_Ks_

$$\text{C}_{\text{1s}}=1-C_{2s}-C{}_{3s}-C{}_{4s}-C_{5s}-C{}_{6s}-C{}_{7s}-C_{8s}-C{}_{9s}-C{}_{10s}-C_{11s}-C{}_{12s}-C{}_{13s}-C{}_{14s}-C{}_{15s}-O_{1s}-O_{2s}$$

$$\frac{\text{dC}_{\text{2s}}}{\text{dt}}=4\alpha_{s}C_{1s}-\beta_{s}C_{2s}+2\beta_{s}C{}_{3s}-3\alpha_{s}C_{2s}+\delta_{s}C{}_{6s}-\gamma_{s}C_{2s}$$

$$\frac{\text{dC}_{\text{3s}}}{\text{dt}}=3\alpha_{s}C_{2s}-2\beta_{s}C_{3s}+3\beta_{s}C{}_{4s}-2\alpha_{s}C_{3s}+\delta_{s}C{}_{7s}-2\gamma_{s}C_{3s}$$

$$\frac{\text{dC}_{\text{4s}}}{\text{dt}}=2\alpha_{s}C_{3s}-3\beta_{s}C_{4s}+4\beta_{s}C{}_{5s}-\alpha_{s}C_{4s}+\delta_{s}C{}_{8s}-3\gamma_{s}C_{4s}$$

$$\frac{\text{dC}_{\text{5s}}}{\text{dt}}=\alpha_{s}C_{4s}-4\beta_{s}C_{5s}+\delta_{s}C{}_{9s}-4\gamma_{s}C_{5s}$$

$$\frac{\text{dC}_{\text{6s}}}{\text{dt}}=2\beta_{s}C_{7s}-3\alpha_{s}C_{6s}+\gamma_{s}C_{2s}-\delta_{s}C{}_{6s}$$

$$\frac{\text{dC}_{\text{7s}}}{\text{dt}}=3\alpha_{s}C_{6s}-2\beta_{s}C_{7s}+3\beta_{s}C{}_{8s}-2\alpha_{s}C_{7s}+2\delta_{s}C{}_{10s}-\gamma_{s}C_{7s}+2\gamma_{s}C_{3s}-\delta_{s}C{}_{7s}$$

$$\frac{\text{dC}_{\text{8s}}}{\text{dt}}=2\alpha_{s}C_{7s}-3\beta_{s}C_{8s}+4\beta_{s}C{}_{9s}-\alpha_{s}C_{8s}+2\delta_{s}C{}_{11s}-2\gamma_{s}C_{8s}+3\gamma_{s}C_{4s}-\delta_{s}C{}_{8s}$$

$$\frac{\text{dC}_{\text{9s}}}{\text{dt}}=\alpha_{s}C_{8s}-4\beta_{s}C_{9s}+2\delta_{s}C{}_{12s}-3\gamma_{s}C_{9s}+4\gamma_{s}C_{5s}-\delta_{s}C{}_{9s}$$

$$\frac{\text{dC}_{\text{10s}}}{\text{dt}}=3\beta_{s}C_{11s}-2\alpha_{s}C_{10s}+\gamma_{s}C_{7s}-2\delta_{s}C{}_{10s}$$

$$\frac{\text{dC}_{\text{11s}}}{\text{dt}}=2\alpha_{s}C_{10s}-3\beta_{s}C_{11s}+4\beta_{s}C{}_{12s}-\alpha_{s}C_{11s}+3\delta_{s}C{}_{13s}-\gamma_{s}C_{11s}+2\gamma_{s}C_{8s}-2\delta_{s}C{}_{11s}$$

$$\frac{\text{dC}_{\text{12s}}}{\text{dt}}=\alpha_{s}C_{11s}-4\beta_{s}C_{12s}+3\delta_{s}C{}_{14s}-2\gamma_{s}C_{12s}+3\gamma_{s}C_{9s}-2\delta_{s}C{}_{12s}$$

$$\frac{\text{dC}_{\text{13s}}}{\text{dt}}=4\beta_{s}C_{14s}-\alpha_{s}C_{13s}+\gamma_{s}C_{11s}-3\delta_{s}C{}_{13s}$$

$$\frac{\text{dC}_{\text{14s}}}{\text{dt}}=\alpha_{s}C_{13s}-4\beta_{s}C_{14s}+4\delta_{s}C{}_{15s}-\gamma_{s}C_{14s}+2\gamma_{s}C_{12s}-3\delta_{s}C{}_{14s}$$

$$\frac{\text{dC}_{\text{15s}}}{\text{dt}}=\eta_{s}O_{1s}-\theta_{s}C_{15s}+\gamma_{s}C_{14s}-4\delta_{s}C{}_{15s}$$

$$\frac{\text{dO}_{\text{1s}}}{\text{dt}}=\theta_{s}C_{15s}-\eta_{s}O_{1s}+\omega_{s}O_{2s}-\psi_{s}O{}_{1s}$$

$$\frac{\text{dO}_{\text{2s}}}{\text{dt}}=\psi_{s}O{}_{1s}-\omega_{s}O_{2s}$$

$$\alpha_{\text{s}}=0.00052e^{(0.21\frac{VF}{RT})}$$

$$\beta_{\text{s}}=0.000235e^{(-0.242\frac{VF}{RT})}$$

$$\gamma_{\text{s}}=0.00725e^{(2.43\frac{VF}{RT})}$$

$$\delta_{\text{s}}=0.00153e^{(-0.626\frac{VF}{RT})}$$

$$\theta_{\text{s}}=0.00096$$

$$\eta_{\text{s}}=0.0167e^{(-1.34\frac{VF}{RT})}$$

$$\psi_{\text{s}}=0.000341e^{(1.24\frac{VF}{RT})}$$

$$\omega_{\text{s}}=0.000262e^{(-0.807\frac{VF}{RT})}$$

Ultrarapidly activating delayed rectifier K^+^ current I_Kur_

$$\text{C}_{\text{1ur}}=1-C_{2ur}-C{}_{3ur}-C{}_{4ur}-I_{ur}-O_{ur}$$

$$\frac{\text{dC}_{\text{2ur}}}{\text{dt}}=4\alpha_{ur}C_{1ur}-\beta_{ur}C_{2ur}+2\beta_{ur}C{}_{3ur}-3\alpha_{ur}C_{2ur}$$

$$\frac{\text{dC}_{\text{3ur}}}{\text{dt}}=3\alpha_{ur}C_{2ur}-2\beta_{ur}C_{3ur}+3\beta_{ur}C{}_{4ur}-2\alpha_{ur}C_{3ur}$$

$$\frac{\text{dC}_{\text{4ur}}}{\text{dt}}=2\alpha_{ur}C_{3ur}-3\beta_{ur}C_{4ur}+4\beta_{ur}O{}_{ur}-\alpha_{ur}C_{4ur}$$

$$\frac{\text{dO}_{\text{ur}}}{\text{dt}}=\alpha_{ur}C_{4ur}-4\beta_{ur}O_{ur}+K_{bur}I{}_{ur}-K_{fur}O_{ur}$$

$$\frac{\text{dI}_{\text{ur}}}{\text{dt}}=K_{fur}O_{ur}-K_{bur}I{}_{ur}$$

$$\alpha_{ur}=e^{(V-48.4)/49.2}$$

$$\beta_{ur}=e^{(V-48.4)/49.2}\frac{e^{-(V+48.4)/13.6}}{1+0.023e^{-(V+48.4)/13.6}}$$

$$\text{K}_{\text{fur}}=0.0001ms^{-1}$$

$$\text{K}_{\text{bur}}=0.0001ms^{-1}$$
